# Striving toward hyperthermia-free analgesia: lessons from loss-of-function mutations of human *TRPV1*

**DOI:** 10.1172/JCI167338

**Published:** 2023-02-01

**Authors:** Tingting Li, Man-Kyo Chung

**Affiliations:** Department of Neural and Pain Sciences, School of Dentistry, University of Maryland, Program in Neuroscience, Center to Advance Chronic Pain Research, Baltimore, Maryland, USA.

## Abstract

Transient receptor potential vanilloid 1 (TRPV1), a receptor for capsaicin and noxious heat, has been one of the most compelling targets for analgesics. However, systemic inhibition of TRPV1 is an impractical approach as a pain killer, since systemic antagonism induces hyperthermia. Two articles in this issue of the *JCI* report phenotypes from separate, rare missense mutations of human TRPV1. He, Zambelli, and colleagues investigated *TRPV1*^K710N^, which showed reduced functionality, while Katz, Zaguri, and co-authors reported on *TRPV1*^N331K^, which led to a complete functional knockout. The findings provide insights that will improve our understanding of the endogenous functions of TRPV1 in humans and may facilitate a rational TRPV1-targeting approach to achieve hyperthermia-free analgesia.

## Hurdles for treating chronic pain through targeting of TRPV1

The sensory system detects noxious stimuli and allows us to avoid harmful tissue-damaging injuries (e.g., withdrawing a hand upon touching a hot stove), which is beneficial for survival. However, persistent pain from pathological conditions, such as chronic inflammation of knee joints or peripheral nerve damage, has no such protective value and needs to be treated. Current primary therapeutics for chronic pain are often limited in their analgesic efficacy and may produce adverse side effects. High addiction liability is a problem of opioidergic medications. An ideal treatment for chronic pain needs to selectively alleviate persistent pain conditions without influencing the beneficial pain sensation and without producing adverse side effects.

Capsaicin, the main ingredient of chili pepper, reliably produces acute burning pain in humans and pain-like behaviors in animals. The discovery of transient receptor potential vanilloid 1 (TRPV1), a receptor for capsaicin (1), opened an era in pain research, which was recognized in 2021 with a Nobel prize to David Julius and Ardem Patapoutian for the discovery of temperature and touch receptors. TRPV1 is a cationic ion channel that can be activated not only by capsaicin, but also by heat, acid, and multiple other exogenous chemicals or endogenous mediators. Now we understand that the burning sensation following the ingestion of capsaicin or exposure to noxious heat is mediated by the activation of TRPV1 expressed in nociceptors, a subset of sensory neurons that respond to noxious stimuli. Functions of TRPV1 are enhanced under inflammatory conditions, and TRPV1 mediates increased pain sensation (hyperalgesia) after tissue injuries. Despite the obvious potential use of TRPV1 inhibitors as pain killers, the systemic pharmacological inhibition of TRPV1 is not practical, since systemic TRPV1 antagonism induces on-target adverse side effects, namely hyperthermia and an increased risk of burn injury by decreasing heat sensation (2). To develop approaches to targeting TRPV1 for alleviation of pathological pain without adverse side effects, highly sophisticated investigations are necessary to reveal the TRPV1 structures associated with in vivo outcomes. A better understanding of the neurobiology and endogenous functions of TRPV1 in humans is critical. The discovery of rare gain-of-function or loss-of-function mutations in *SCN9A*, a voltage-gated sodium channel gene encoding Nav1.7, revealed its critical role in nociceptors (3). While mild phenotypes associated with common TRPV1 genetic variants are known (4), our understanding of the phenotypic effects of rare TRPV1 mutations is limited. Complete or partial insensitivity to capsaicin, which might be attributable to the reduced expression of TRPV1, has been reported (5, 6). However, genomic mechanisms involving TRPV1 mutations leading to the phenotypes are inconclusive. While such information should increase our understanding of the pathophysiological roles of TRPV1 in humans, developing analgesic strategies through selective targeting of TRPV1 function in humans without adverse side effects is another challenge. Two articles published in this issue of the *JCI* shed light on overcoming these hurdles.

## TRPV1-targeted, hyperthermia-free inhibition of pathological pain

**Figure 1:**
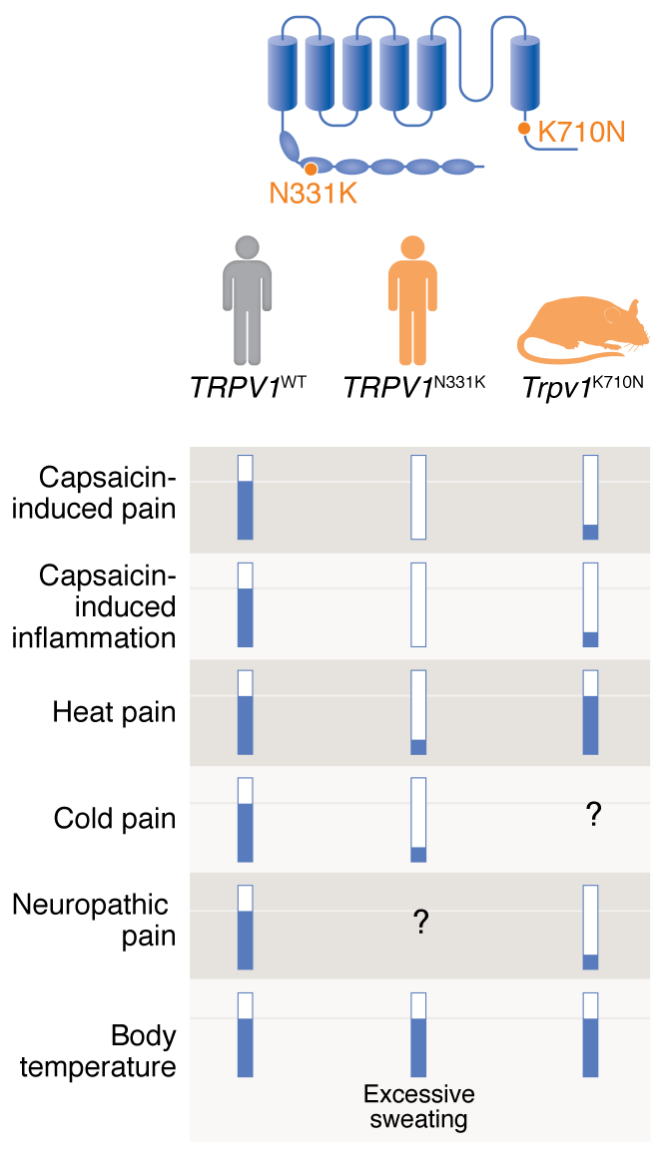
Phenotypes of two rare loss-of-function missense mutations of human TRPV1. Katz, Zaguri, and co-authors (9) reported phenotypes of two individuals carrying *TRPV1*^N331K/N331K^, which is located in the ankyrin repeat domain at the N-terminal domain. He, Zambelli, and co-authors (7) reported phenotypes of knockin mice carrying *Trpv1*^K710N/K710N^, a loss-of-function missense mutation rarely observed in humans. TRPV1^K710N^ is located in the TRP domain of the C-terminal domain. *TRPV1*^N331K/N331K^ shows a complete deficit in activation, whereas TRPV1^K710N^ shows a partial loss of function.

He, Zambelli, and co-authors (7) report on phenotypes of a rare human *TRPV1* mutation through reverse translation using a mouse line carrying the ortholog missense mutation. Through a clever in silico approach comparing human TRPV1 rare mutations with avian TRPV1 lacking capsaicin sensitivity, the authors attempted to identify a single residue that eliminates capsaicin sensitivity without altering heat sensitivity or thermoregulation. *TRPV1*^K710N^ is an extremely rare mutation in humans. The mutation induces structural alteration in the TRP domain that is critical in channel gating and multimerization (8). Cells carrying *TRPV1*^K710N^ exhibited greatly reduced TRPV1 activation in response to capsaicin in vitro (Figure 1). When the K710-to-N710 mutation of the orthologous TRPV1 residue was created in mice, neurogenic inflammation or capsaicin-induced behavioral responses were reduced by half. Importantly, heat sensitivity in the tail was not affected. Strikingly, *Trpv1*^K710N^ mice showed a substantial decrease in thermal hyperalgesia and a lack of mechanical hyperalgesia after nerve injury. He, Zambelli, and co-authors further extended the findings and developed a method for targeting the region involving K710 using V1-cal, a membrane-permeable peptide composed of ten amino acids of TRPV1 (R701 to S711) and ten TAT sequences. The V1-cal peptide reduced capsaicin-induced pain-like behaviors and neurogenic inflammation in vivo. Systemic administration of V1-cal peptide via an osmotic pump reversed neuropathy-induced thermal, mechanical, and cold hyperalgesia. In both *Trpv1*^K710N^ mice and V1-cal–treated naive mice, body temperature was not affected. Although He, Zambelli, and colleagues were unable to validate these *TRPV1*^K710N^ phenotypes in humans, preclinical data suggest the potential for a hyperthermia-free analgesic approach using a peptide targeting a narrowly defined region of TRPV1.

## Individuals lacking functional TRPV1

In studies of individuals carrying another rare TRPV1 mutation (*TRPV1*^N331K^), Katz, Zaguri, and co-authors (9) report on phenotypes resulting from the complete loss of function of TRPV1 (Figure 1). Individuals with the functional knockout commonly carried the rare homozygous missense mutation of *TRPV1*^N331K^. The mutation is located in the ankyrin repeat domain, which is critical for channel activation (10). In vitro assays showed that *TRPV1*^N331K^ was not activated by capsaicin or other agonists. The expression and targeting of TRPV1^N331K^ to the plasma membrane were normal, suggesting that the mutation selectively affects the function of TRPV1, likely through interference with channel gating. Consistently, the homozygous, but not heterozygous, carriers of *TRPV1*^N331K^ lacked aversion to capsaicin and showed no pain or flare in the skin upon injection of capsaicin. The affected individuals showed a modest reduction in heat pain. Heterozygous *TRPV1*^N331K/+^ carriers did not show such phenotypes (9). These findings are consistent with the phenotypes of mice lacking the expression of TRPV1 or humans treated with a TRPV1 antagonist (2). Further genetic analysis of the affected individuals and their family strongly suggested that the phenotypes are primarily derived from the missense mutation of *TRPV1*. Although another homozygous missense mutation in *PROKR1*, which is also implicated in pain, was identified in the family, two individuals carrying the homozygous mutation of *PROKR1* with heterozygous *TRPV1*^N331K/+^ did not show phenotypes comparable to those of the *TRPV1*^N331K/N331K^ carriers (9). Therefore, the findings convincingly support the contribution of *TRPV1* to chemical and thermal pain in humans.

## Lessons for hyperthermia-free analgesia

The results from Katz, Zaguri, and colleagues (9) suggest species differences in the roles of TRPV1 and help us to anticipate the potential effects of TRPV1 antagonism in humans. The homozygous carrier of *TRPV1*^N331K/N331K^ showed a reduction in cold pain (9), which is consistent with the necessity of TRPV1 for cold nociception and cold hyperalgesia in rodents (11). Therefore, TRPV1 antagonism possibly influences the protection from both noxious heat and cold. In the carrier homozygous for *TRPV1*^N331K/N331K^, pain and flare induced by allyl isothiocyanate (mustard oil), an agonist of transient receptor potential ankyrin subtype 1 (TRPA1), was increased (9). This finding is reminiscent of a report of an individual carrying mutations in the introns of *TRPV1* who is completely insensitive to capsaicin and exhibits increased sensitivity to a garlic extract, another TRPA1 activator (5). This phenomenon may be attributable to the interactions of TRPA1 and TRPV1 in sensory neurons (12). However, there is no evidence that in vivo responses to the activation of TRPA1 are greater in *TRPV1*-knockout mice, suggesting that species-specific mechanisms regulate TRPV1-TRPA1 interactions. Since TRPA1 is a sensor of multiple environmental irritants (13), TRPV1 antagonism potentially increases sensitivity to chemical irritation in humans. In contrast, histamine-mediated itch and skin flare were not altered in the affected individual (9), which is consistent with the lack of effect of a TRPV1 antagonist on histamine-induced itch in healthy volunteers (14). Considering the profound involvement of TRPV1 with itch in rodents (15), there is an apparent gap in pruriceptive mechanisms between mice and humans.

Although *TRPV1*^N331K/N331K^ carriers had a normal core body temperature, they showed excessive sweating, which might be a compensatory response to their lifelong deficiency in TRPV1-mediated body temperature regulation (9). This finding might suggest that the disturbance of thermoregulation by TRPV1 antagonism is physiologically adaptable, which is consistent with the observation that repeated administration of TRPV1 antagonists attenuates hyperthermia (16, 17).

Unlike with conventional TRPV1 antagonists, He, Zambelli, and co-authors reported that systemic administration of V1-cal did not produce hyperthermia in mice, suggesting that the region-specific targeting of TRPV1 can lead to potential hyperthermia-free analgesia (7). Further determination of mechanisms through which partial antagonism by V1-cal produces robust analgesia without altering body temperature would strengthen the study’s translational value. It would be critical to determine the efficacy of V1-cal in inhibiting TRPV1 upon polymodal activation, since hyperthermia mediated by a TRPV1 antagonist depends on its inhibition of the activation by acid but not by heat or capsaicin (18). The outcomes may guide the design of small molecules relating to TRPV1^K710^. It will also be important to determine the effects of V1-cal on baseline pain-like responses to noxious heat, cold, and mechanical stimuli in vivo. Mechanisms of TRPV1 inhibition by V1-cal are puzzling. A previous study shows that a membrane-tethered peptide targeting different residues within the TRP domain interferes with TRPV1 subunit association and decreases inflammatory pain (19). Any off-target effects of V1-cal also need to be carefully assessed (e.g., studying V1-cal effects in *TRPV1*-KO mice).

## Conclusion

TRPV1 has been one of the most compelling targets for the development of analgesics. Topical capsaicin, a TRPV1 agonist, is approved for treating chronic neuropathic pain (20), and it is fascinating to navigate the potential strategies for the treatment of chronic pain conditions using both TRPV1 agonists and antagonists. A better understanding of modality-specific TRPV1 functions and antagonism should expedite the development of a class of TRPV1 antagonists and achieve hyperthermia-free analgesia.

## Version 1 02/01/2023

Electronic publication
